# Understanding the Role of Chemerin in the Pathophysiology of Pre-Eclampsia

**DOI:** 10.3390/antiox12040830

**Published:** 2023-03-29

**Authors:** Katarzyna Pankiewicz, Tadeusz Issat

**Affiliations:** Department of Obstetrics and Gynecology, Institute of Mother and Child in Warsaw, Kasprzaka 17a, 01-211 Warsaw, Poland

**Keywords:** chemerin, pre-eclampsia, oxidative stress, endothelial dysfunction

## Abstract

Chemerin is a multifaceted adipokine that is involved in multiple biological processes, including inflammation, angiogenesis, adipogenesis, and energy metabolism, as well as oxidative stress. There is a vast body of evidence for a crucial role of chemerin in the development of different cardiovascular diseases. Blood chemerin levels, as well as its placental expression, are elevated in patients with pre-eclampsia (PE) and correlate positively with the severity of the disease. This narrative review summarizes the current knowledge about the potential role of chemerin during PE development, with a particular focus on its involvement in oxidative stress and endothelial dysfunction.

## 1. Introduction

Adipokines are autocrine and paracrine factors that are released from human adipose tissue, possessing multiple biological properties (i.e., acting as pro-inflammatory cytokines, growth factors, and anti- or pro-oxidants) and being involved in the pathogenesis of diabetes, hypertension, metabolic syndrome, and other cardiovascular diseases [1]. The first discovered adipokine was leptin, which was identified in 1994 [2]. Since that time, plenty of adipokines have been discovered [3].

Chemerin is a multifaceted molecule that was originally discovered as a novel retinoic acid-receptor responder 2 (RARRES2) gene in psoriatic skin lesions, implying an immunomodulating role [4]. Chemerin is an adipokine that is involved in inflammation, angiogenesis, adipogenesis, and energy metabolism, as well as in oxidative stress [5]. There is a vast body of evidence for a crucial role of chemerin in the development of different cardiovascular diseases [6]. Blood chemerin levels, as well as its placental expression, are elevated in patients with pre-eclampsia (PE) and correlate positively with the severity of the disease, suggesting its important role in PE pathophysiology [7]. 

According to The International Society for the Study of Hypertension in Pregnancy (ISSHP), the definition of PE includes a new-onset hypertension that occurs after 20 weeks of gestation, together with proteinuria or acute kidney injury, liver failure, neurological symptoms, hemolysis or thrombocytopenia, or fetal growth restriction (FGR) [8]. PE is diagnosed at an incidence of 3-8% of all pregnancies and remains the main cause of perinatal morbidity and mortality in developed countries [9]. Two types of PE can be distinguished: early-onset PE developing before 34 weeks of gestation and late-onset PE occurring after 34 weeks of gestation [8]. The risk factors for developing PE include family history of PE or PE in previous pregnancies, first pregnancy, multiple pregnancy, maternal age >40 years, pregnancy after assisted reproduction techniques, obesity, black race, and chronic maternal diseases, such as hypertension, diabetes type 1 or 2, chronic kidney disease, systemic lupus erythematosus, antiphospholipid syndrome, and inherited thrombophilia [10]. There is a well-evidenced relationship between PE and an increased risk of cardiovascular and kidney disease in the patient’s future life but also in the offspring of mothers with PE [11,12,13]. The pathophysiology of PE is very complex and is currently intensively studied, and this study is aimed at elaborating modern predictive and therapeutic strategies that are dedicated to women at an increased risk of developing PE to improve maternal and fetal outcomes. 

The aim of this narrative review was to summarize the available knowledge about the potential role of chemerin in the pathophysiology of PE, with a particular focus on its involvement in oxidative stress and endothelial dysfunction.

## 2. Chemerin Structure and Biological Functions

Chemerin is encoded by the *TIG2* gene (tarazotene-induced gene 2), which is also known as RARRES2, and it was firstly described in 2003 as a ligand for an orphan G-protein-coupled receptor (GPCR), namely, chemokine-like receptor 1 (CMKLR1), which is also known as chemerin receptor 1 or ChemR23 [4,14,15]. In humans, chemerin is highly expressed in a wide range of tissues: endocrine glands (such as adrenal gland or parathyroid), liver, pancreas, lungs, kidneys, adipose tissue, and female reproductive organs, including placenta [16]. Firstly, chemerin function was described as acting like a chemokine by recruiting CMKLR1-expressing leukocytes to the inflammatory site [14]. Further research revealed that chemerin is highly expressed in adipocytes and regulates adipocyte metabolism, suggesting that it should be treated as a novel adipokine [17,18]. At least 6 chemerin isoforms with different biological activity are known, and their secretion is dependent on the C-terminal proteolysis of chemerin precursors: pre-pro-chemerin (163 amino-acids protein) and pro-chemerin (143 amino-acids protein) [19]. The average plasma level of chemerin in the population is approximately 50ng/mL [6,20].

Chemerin acts via 3 different receptors, including the abovementioned CMKLR1, G-protein-coupled receptor 1 (GPR1, chemerin receptor 2), and chemokine CC-motif receptor-like 2 (CCRL2, chemerin receptor 3) [19]. CMKLR1 is the main chemerin receptor. It activates the Gαi and Gαo proteins, leading to a strong signal response to chemerin: intracellular Ca2+ release, suppression of cyclic adenosine 3’5’ monophosphate (cAMP) accumulation, and phosphorylation of extracellular signal-regulated kinase 1/2 (ERK1/2). The chemerin/CMKLR1 axis promotes chemotaxis of natural killers (NK), macrophages, and dendritic cells, as well as cell proliferation. The binding of chemerin to GPR1 results in a weak Ca2+ mobilization and phosphorylation of ERK1/2, but this action participates in the anti-inflammatory response, development of adiposity, hormone secretion, and the regulation of glucose balance in obesity, as well as in the regulation of angiogenesis [21,22]. Both CMKLR1 and GPR1 activation can lead to the recruitment of β-arrestins and receptor internalization [23,24]. GPR1 serves additionally as a scavenger receptor for peptides that cannot stimulate receptor activation. Interestingly, GPR1 is not expressed in monocytes, macrophages, or peripheral blood lymphocytes, but instead, it is expressed in cells related to the central nervous system [24,25]. Although chemerin is the only identified ligand for CCRL2, its interaction does not initiate any known signaling pathway; thus, its role remains uncertain; however, it seems to have the ability to amplify local chemerin concentration for CMKLR1 interaction [19,26]. 

The main biological functions of chemerin are its actions as an adipokine, chemoattractant, and growth factor [25]. As an adipokine, chemerin regulates glucose and lipid metabolism, affecting, e.g., lipid deposition in the endothelium, resulting in the progression of atherosclerosis and the development of insulin resistance and obesity [17,27]. As a chemoattractant for various leukocyte populations, including macrophages, dendritic cells, and NK cells, it may serve as a pro- or anti-inflammatory factor depending on the context and the disease model [5,28]. A very important role of chemerin is the regulation of angiogenesis, and this molecule can be recognized as a stimulator of angiogenesis; however, there are also some available studies revealing chemerin as an anti-angiogenic factor and a strong vasoconstrictor, participating in blood pressure control and the development of endothelial dysfunction [29,30]. Chemerin is also known as a growth factor that is involved in the regulation of microvasculatory vessel growth and adipocyte aggregation, as well as inosteoblastogenesis of bone-marrow-derived precursor cells [31]. Recent studies have suggested that chemerin plays an important role in cancer; it can be reduced or upregulated in different cancer tissues, presenting protective or stimulating effects on carcinogenesis. The anti-tumoral effect of chemerin includes mitogen-activated protein kinase (MAPK), β-catenin and protein kinase B (AKT) inhibition, and phosphatase and tensin homolog (PTEN) activation, whereas the cancer-promoting effects are mediated by the activation of matrix metalloproteinases (MMPs), MAPK, p38, and AKT, with the involvement of β-arrestin-1 [26]. 

In light of the wide range of biological chemerin functions presented here, recent research has revealed that this molecule is involved in the pathophysiology of many human diseases, such as obesity, diabetes, metabolic syndrome, cardiovascular diseases, cancer, liver failure, chronic pancreatitis, chronic kidney disease, and psoriasis [5,6,17,25,26]. In this review, we try to explain how different biological chemerin activities might be involved in PE pathophysiology. 

## 3. Key Points of PE Pathophysiology

The classical understanding of PE pathophysiology is a two-stage disease model that was introduced by Redman in 1991 and was updated in the following years [32,33,34]. Stage 1 is confined to the placenta; it is caused by inadequate trophoblast invasion of the maternal spiral arteries that leads to reduced placental perfusion. Stage 2 is the onset of maternal symptoms due to the release of numerous biological factors from the ischemic placenta, causing endothelial dysfunction [32]. The critical points in the first stage of PE are the abnormal remodeling of uterine arteries and syncytiotrophoblast (STB) stress. In normal pregnancy, extra villous trophoblast (EVT) invades the spiral arteries, displacing the vascular smooth muscle and endothelial cells. In consequence, spiral arteries change from high-resistant to low-resistant vessels that are able to ensure appropriate placental perfusion [35]. When this process is disturbed, the placenta is unable to provide sufficient blood flow for the developing fetus, leading to fetal growth restriction and STB stress [36,37]. Multiple factors that are shed by the hypoxic placenta have been identified as connecting the two stages of disease, including 1. pro-inflammatory cytokines: tumor necrosis factor α (TNFα) and interleukin 1 and 6 (IL-1, IL-6); 2. angiogenic and anti-angiogenic factors: placental growth factor (PlGF), soluble fms-like tyrosine kinase 1 (sFlt-1), and soluble endoglin (sEng); 3. other factors: hypoxia-induced factor 1 (HIF-1), endothelin-1, extracellular vesicles (ECVs), and angiotensin II 1 receptor autoantibodies (AT1-AA); and 4. oxidative stress and endoplasmic reticulum (ER) stress [38,39,40]. Via different mechanisms and signaling pathways, all these factors cause endothelial dysfunction in the mother, manifesting as hypertension, proteinuria, kidney or liver failure, and cerebrovascular incidents. The anti-angiogenic state and oxidative stress play a particular role in this process, and they are described below. 

It needs to be highlighted that maternal diseases, such as obesity, chronic hypertension, and diabetes, are well-known risk factors for PE because they are related to chronic vascular inflammation. They probably affect both the periconceptual endometrial function and spiral artery remodeling. Furthermore, maternal vascular inflammation may cause an excessive response to even a relatively small degree of STB stress, leading to the clinically overt stage 2 [41,42]. Redman and Staff point out that pregnancy is a “race against time”, in which delivery will save many pregnancies from developing PE, even in pregnancies that are normotensive until term; if the pregnancy lasts longer, because of the ageing placenta, late-onset PE could be revealed, leading to fetal demise and maternal complications [33,34,38]. Maternal factors may impact both stages of disease, because they affect both placental function and maternal vulnerability to factors that are shed by placental tissue and are responsible for the onset of clinical symptoms [34].

### 3.1. Oxidative Stress

The term “oxidative stress” was introduced in 1985 by Cadenas and Sies and is defined as an imbalance between oxidants and antioxidants with the advantage of reactive oxygen species (ROS), leading to abnormal redox signaling and cellular damage [43]. This process is crucial in linking two stages of PE. Reduced placental perfusion due to impaired trophoblastic invasion triggers excessive ROS production that cannot be antagonized by antioxidants because of their decreased activity [44]. The main ROS and antioxidants involved in oxidative stress during PE are presented in Table 1. There is also a parallel process known as nitrosative stress, which is defined as an imbalance between nitrosants (RNS reactive nitrogen species) and antioxidants. The most important RNS are nitric oxide (•NO) and peroxynitrite (ONOO^−^) [45]. The currently available research suggests that during PE, oxidative stress is induced by placental malperfusion, with repeated hypoxia and reoxygenation stimulating the activation of xanthine oxidase and nicotinamide adenine dinucleotide phosphate (NADPH) oxidase (Nox) [46,47]. Oxidative stress can be also exacerbated due to increased serum levels of TNFα. TNFα acts directly on the activation of NADPH oxidase but also indirectly by up-regulating lectin-like oxidized low-density lipoprotein (LDL) receptor-1 (LOX-1), which contributes to the increased uptake of oxidized LDL and O_2_•^−^ production [46]. Other factors circulating in the maternal blood during PE including pro-inflammatory cytokines (IL-6), AT1-AA, and free fetal hemoglobin (HbF) may also intensify oxidative stress and endothelial dysfunction [47]. 

Another term that is directly related to oxidative stress is ER stress. The ER is an intracellular structure that is responsible for the correct conversion of various proteins and the control of their folding. About 30% of total placental oxygen consumption is used for the oxidative process of protein folding. In pathological conditions, e.g., due to hypoxia, the reaction that is named unfolded protein response (UFR) is triggered. UFR involves the inhibition of protein folding in order to reduce the synthesis of these proteins in the absence of the proper supply of oxygen and nutrients. Moreover, affected cells may undergo apoptosis, and if it concerns trophoblast cells, large amounts of STB microparticles are released into the maternal blood and stimulate the inflammatory response [48,49,50].

It needs to be highlighted that during PE, oxidative stress and ER stress affect both the placenta (STB stress) and systemic maternal vasculature, leading to endothelial dysfunction. STB is more vulnerable to oxidative stress than other tissues because of the insufficient concentration of antioxidants such as manganese superoxide dismutase (MnSOD), as well as an increased sensitivity to ROS resulting from the presence of abundant unsaturated fatty acids (target for ROS) in the plasma membranes of STB cells [51,52]. The most important molecular consequences of oxidative stress in PE leading to endothelial dysfunction and maternal systemic disease are lipid peroxidation, protein carbonylation, and DNA damage. Oxidative stress can also induce the accumulation of advanced oxidative protein products (AOPPs)—causing trophoblast cell damage and promoting sFlt-1 production, as well as advanced glycation end products (AGEs)—activating several signaling pathways and leading to an inflammatory process [46,47]. It is noteworthy that during PE, endothelial dysfunction is also a result of the reduced bioavailability of nitric oxide (NO). This, in turn, is a consequence of three main processes: (1) O_2_∙− captures NO for the formation of ONOO^−^ with high redox potential; (2) the increased production of arginase, an enzyme catalyzing the conversion of L-arginine to L-ornithine and urea; (3) the presence of dimethylarginine (ADMA), an inhibitor of endothelial nitric oxide synthase (eNOS) that is able to decrease the synthesis of NO [46,53].

**Table 1:** The most relevant oxidants and antioxidants involved in oxidative stress during PE [46,47,51,52].

Oxidants—Reactive Oxygen Species (ROS)	Antioxidants
Superoxide O_2_^●−^ Hydrogen peroxide H_2_O_2_ Hydroxyl radical HO^●^	ENZYMATIC Superoxide dismutase (SOD) Hemoxygenase (HO-1) Catalase (CAT) Glutathione peroxidase (GPX) Glutathione-S-transferase (GSX)
The main sources of ROS are: NO synthases (NOS) NADPH oxidases (NOX) Mitochondrial electron transport chain (ETC) Xanthine oxidase (XO)	NON-ENZYMATIC Glutathione (GSH) Vitamin C and E Nicotinamide adenine dinucleotide (NADH) Nicotinamide adenine dinucleotide phosphate (NADPH) Transferrin Selenium Melatonin (5-methoxy-N-acetyltryptamine)

Oxidative stress may also alter the chromatin structure and induce nucleic acid damage. Pre-eclamptic placentas with high levels of the oxidative stress marker malondialdehyde (MDA) display an increase in DNA fragmentation that can lead to trophoblast apoptosis [47,54]. Moreover, oxidative stress impacts gene regulation via the epigenetic modulation of fetal programming. This is a probable link between PE and increased cardiovascular risk in adult life in offspring [51,55].

### 3.2. Anti-Angiogenic State

The anti-angiogenic state, which consists of an imbalance between angio- and anti-angiogenic factors, with a shift in favor of the latter, is currently one of the best-documented elements of PE pathophysiology [39,40,49,56]. This disproportion manifests as an increase in the serum level of the main anti-angiogenic factor, sFlt-1, and a decrease in the serum level of the main angiogenic factor, PlGF, and it can be expressed as the ratio of these concentrations, that is, the sFlt-1/PlGF ratio [57,58,59]. 

PlGF is a member of the vascular endothelial growth factor (VEGF) family and is predominantly produced in the placental tissue. It binds to the VEGF type 1 receptor (VEGFR-1) and its soluble form, sFlt-1, but does not bind to the type 2 VEGF receptor (VEGFR-2). The main function of PlGF is to stimulate angiogenesis directly through the VEGFR-1 receptor, as well as indirectly through the enhancement of the VEGF pro-angiogenic effect. Although PlGF does not act through the VEGFR-2, it causes receptor phosphorylation and, thus, increases its sensitivity to circulating VEGF [60,61]. The reduction in the serum PlGF level in the course of PE is due to two reasons: the first is excessive binding to sFlt-1, and the second is the reduction in its production in STB. The latter is an example of a negative response to stress, in this case hypoxia, which was confirmed in experimental studies [38,39].

sFlt-1 as a soluble form of the VEGFR-1 receptor binds both PlGF and VEGF, thereby inhibiting VEGF-dependent angiogenesis. In pre-eclamptic patients, an increased production of sFlt-1 in placental tissue, as well as a significantly higher serum sFlt-1 level, can be detected. The secretion of sFlt-1 is a typical example of a positive stress response and is stimulated by factors such as hypoxia, oxidative stress, TNFα, or angiotensin II [39,62]. The overexpression of sFlt-1 can be observed in trophoblasts early in pregnancy and is associated with abnormal remodeling of the maternal spiral arteries. It can be detected in various conditions that predispose women to the development of PE, such as nulliparity, multiple pregnancy, diabetes mellitus, or chronic arterial hypertension [63,64]. The role of anti-angiogenic factors in the development of PE has been confirmed in animal models. There is evidence that sFlt-1 causes an increase in blood pressure by intensifying the production of endothelin-1 [65]. The administration of exogenous sFlt-1 to pregnant rodents causes a set of symptoms that are typical for PE. On the other hand, the use of VEGF-blocking therapies (e.g., bevacizumab: anti-VEGF monoclonal antibody in the treatment of cancer) is associated with side effects such as hypertension and proteinuria [66,67,68,69,70].

Another important anti-angiogenic factor is sEng. It is a cell surface co-receptor for TGFβ1 and TGFβ3 that influences vascular endothelial function. Significantly higher serum sEng levels are found in patients with PE [48]. Recent studies have suggested that the anti-angiogenic effect of sEng may be mediated by agonizing the bone morphogenic protein 9, an anti-angiogenic protein that controls vascular quiescence in the adult vasculature, as well as by interference with NO mediated vasodilation [71,72].

## 4. Potential Role of Chemerin in the Pathophysiology of PE

Many studies have demonstrated significantly elevated serum chemerin levels, as well as its increased expression in placental tissue in patients with PE. In a study performed by Stepan et al., maternal chemerin serum concentrations were significantly higher in PE patients as compared to healthy controls (249.5 vs. 204.8 μg/L; *p* < 0.001). Additionally, chemerin serum levels positively correlated with blood pressure, creatinine, free fatty acids, cholesterol, triglycerides, leptin, adiponectin, and C-reactive protein (CRP). Six months after delivery, chemerin serum levels remained significantly higher in former PE patients in comparison to healthy controls (196.0 vs. 152.2 μg/L) [73]. A similar case-control study was published by Duan et al., which presented increased serum chemerin levels in women with PE as compared to healthy pregnant women (258.85 ng/mL vs. 210.80 ng/mL; *p* < 0.001) [74]. Xu et al. investigated first-trimester maternal serum chemerin levels in 518 patients and revealed that they were significantly elevated in women who developed PE compared with those without PE and also in patients who developed severe PE compared with mild PE [75]. Another study performed by Cetin et al. confirmed significantly higher serum chemerin levels in severe PE compared to the mild form of the disease (394.72 vs. 322.11 ng/mL) and healthy pregnant women (199.96 ng/mL; *p* = 0.001). In this study, chemerin serum levels were also positively correlated with systolic and diastolic blood pressure, the CRP homeostasis model assessment of insulin resistance (HOMA IR), proteinuria, aspartate transaminase (AST), alanine transaminase (ALT) and duration of hospitalization. Gestational week at delivery, birthweight, and APGAR scores at 1 and 5 min were, in turn, negatively correlated with maternal serum chemerin level. The authors concluded that a maternal serum chemerin level of >252.0 ng/mL indicated pre-eclampsia with 95.5% sensitivity and 95.7% specificity [76]. In a systematic review published in 2020, among various evaluated adipokines, only increased serum levels of leptin, chemerin, and fatty acid-binding protein-4 were associated with PE when measured during both the 1st and 3rd trimester [77]. Turgut et al. investigated the roles of various adipokines in obese and non-obese pre-eclamptic patients and found significantly higher chemerin levels in obese pre-eclamptic women as compared to non-obese pre-eclamptic women and normotensive control group [78]. 

Placental tissue shows a high expression of chemerin and its receptors. Chemerin mRNA and protein were detected in high amounts in the primary stromal cells (STs) and EVTs, but not in decidual endothelial cells (DEC) that were derived from the first-trimester decidual tissues [79]. Additionally, chemerin was also observed in cytotrophoblasts (CTBs), Hofbauer cells, and vascular endothelial cells of human placental tissue derived from the third trimester of pregnancy [80]. Up-regulation of chemerin expression in PE was confirmed in placenta that was derived from the PE-like rat/mice model, as well as from human placenta [7,81]. Tan et al. demonstrated that placental trophoblast chemerin overexpression in mice induced a PE-like syndrome, including hypertension, proteinuria, diminished trophoblast invasion, and up-regulation of sFlt-1 and the inflammation markers nuclear factor kappa B (NFκB), TNFα, and IL-1β. In patients with PE, both serum chemerin levels and its placental expression were increased. Moreover, chemerin serum level was positively correlated with sFlt-1/PlGF ratio [7].

### 4.1. Chemerin during Early Pregnancy, Placentation, and Uterine Artery Remodeling

Successful pregnancy depends on the proper formation of the placenta. The available studies have demonstrated that chemerin is one of the factors involved in normal placentation and remodeling of placental blood vessels. Yang et al. found that decidual chemerin expression in women who had experienced early spontaneous abortion was lower than in those who had experienced normal early pregnancy, whereas CMLKR1 expression was higher in the former than in the latter. The authors also demonstrated in a pregnant mouse model that the intrauterine injection of CMKLR1 receptor antagonist 2-α-naphthoyl ethyltrimethylammonium iodide (α-NETA) caused the inhibition of the chemerin/CMKLR1 signaling pathway, which can lead to the abortion of mouse embryos [82]. 

Trophoblast invasion and uterine spiral artery remodeling are critical moments in normal pregnancy. Normal uterine artery remodeling takes place in two stages: 1. trophoblast-independent remodeling with the participation of uterine NK cells and macrophages that infiltrate the arterial wall via the activation of MMPs; 2. trophoblast-dependent remodeling by which interstitial trophoblast replaces the endothelium [37,83]. Tan et al. studied the consequences of placental chemerin overexpression in pregnancy by using a mouse model. They demonstrated that there was no difference in implantation rates between overexpressed chemerin and the control group, but the pregnancy loss rate was significantly higher in the studied group in comparison to the controls, whereas placental and fetal weight were significantly reduced. Moreover, trophoblast-specific chemerin overexpression induced pre-eclampsia-like symptoms including hypertension, proteinuria, and glomerular endotheliosis in histopathological kidney examination. Hematoxylin and eosin staining of the placenta revealed disorganization in the junction zone and labyrinth layer. Further investigations that used HTR8/Svneo cells overexpressing chemerin showed down-regulation of phosphoinositide 3-kinases (PI3K), Akt, and VEGF-A via CMLKR1 and up-regulation of sFlt-1, NFκB, TNF-α, and IL-1β. The rates of trophoblast migration and invasion were significantly reduced [7]. These findings, together with increased placental chemerin expression and the strong positive correlation between serum chemerin levels and the sFlt-1/PlGF ratio in pre-eclamptic women, indicate an important role of chemerin in abnormal placentation in PE and support the concept of the placenta being a main source of maternal chemerin. 

Zhang et al. confirmed CMKLR1 expression in the decidual NK cells and chorionic villi of the human placenta. They also found that chemerin suppressed the proliferation of the decidual NK cells in vitro. Additionally, in the Cmklr1-knockout mice model, the authors showed that the Cmklr1 deficiency negatively affected implantation and contributed to the development of FGR. The Cmklr1-knockout mice presented histological changes indicating an increased diameter of spiral arteries and increased trophoblast invasion in the decidua but also an increased number of NK cells in the placental tissue, as well as an elevated serum IL-15 level, which is known for playing an important role in the development, survival, and activation of NK cells. On the basis of these findings, the authors concluded that chemerin and CMKLR1 are involved in the regulation of the secretion of trophoblast regulatory factors from decidual NK cells, including TNF-α, IL-10, and IL8, affecting in this way placental development and spiral artery remodeling [84]. The other study group demonstrated high levels of CMKLR1 expression by the CD56^low^ peripheral blood NK (pbNK) cells and observed that chemerin significantly supports pbNK cell migration through DEC and ST cells, as well as dNK cell migration through ST cells via rapid ERK activation. A very important finding of this study is evidence indicating that DEC express the chemerin receptor CMKLR1, and that once exposed to chemerin, they undergo capillary-like tube formation, thus suggesting that chemerin participates also in decidual vessel remodeling [79]. Ji et al. in their study revealed that chemerin, by activating the CMKLR1/Akt/CCAAT-enhancer-binding protein α (CEBPα) axis, promotes M1 macrophage polarization, suppresses trophoblast migration, invasion, and angiogenesis, thus contributing to PE development [85]. In the pre-eclamptic placenta, macrophages shift from the M2 to M1 phenotype. This shift is accompanied by an increase in pro-inflammatory cytokines (such as TNF-α, IL-6, and IL-8) and a decrease in anti-inflammatory cytokines (such as IL-10). Abnormal macrophage activation leads to production of various molecules (such as TNF-α and interferon γ IFN-γ) that may affect trophoblast invasion [86].

### 4.2. Pro-Inflammatory Effect

PE is associated with pro-inflammatory status—inflammation affecting both trophoblast and maternal vasculature. Placental injury as a result of hypoxia/reperfusion syndrome is associated with a pathological immune reaction and can trigger a systemic inflammatory response and endothelial damage [87,88]. Chemerin is a well-known chemoattractant and enables the interaction of macrophages with dendritic cells and NK, directing them toward locations where damage occurs [14,28]. It is particularly important in relation to NK involvement in spiral artery remodeling, as described earlier. Quan et al. investigated chemerin regulation of pyroptosis and trophoblast inflammation in PE. Using hypoxia/reoxygenation challenged human and rat trophoblast, as in the in vitro model, they demonstrated that hypoxia/reoxygenation upregulates chemerin expression through homeobox A9 (HOXA9), and that in turn, chemerin enhances hypoxia/reoxygenation induced pyroptosis of trophoblasts through CMLKR1/AMP-activated protein kinase (AMPK)/thioredoxin-interacting protein (TXNIP)/NOD-like receptor pyrin-containing receptor 3 (NLRP3) inflammasome pathway [81]. In other studies, HOXA9 and ephrin type-B receptor 4 (EPHB4) expression were increased in pre-eclamptic placenta, and HOXA9 promoted EPHB4 expression and impaired the migration and invasion of HTR-8/Svneo cells [89]. Pyroptosis is an inflammatory, programmed cell death that was firstly described in macrophages infected with Salmonella [90]. Pyroptosis is characterized by NLRP3 inflammasome-promoted and caspase-1-dependent plasma membrane rupture and the release of damage-associated molecular patterns (DAMPs) and cytokines such as IL-1β and IL-18 into the extracellular matrix, leading to sterile inflammation, one of the key mechanisms in PE development [91,92,93,94].

There is evidence that chemerin may enhance the expression of various inflammatory factors, such as IL-6, TNFα, and CRP, resulting in blood vessel wall inflammation, increased monocyte attachment to endothelial cells, and endothelial dysfunction. Moreover, TNFα, IL-1β, and IL-6 were demonstrated to increase the expression of the CMKLR1 receptor in endothelial cells. In human coronary artery endothelial cells, chemerin is associated with the overexpression of intercellular adhesion molecule 1 (ICAM-1) and E-selectin, which are typical markers of vascular endothelial activation [6,95,96,97]. In patients with coronary artery disease, serum chemerin levels are correlated with inflammatory markers, such as white blood cell (WBC) count, high-sensitivity CRP (hsCRP), platelet count, fasting insulin, and c-peptide [98]. Chemerin was also found to induce NF-κB activation via the MAPK and PI3K/Akt pathways [99]. NF-κB is an important regulator of embryo implantation, the remodeling of spiral arteries, and the development of the placenta. NF-κB regulates the production of pro-inflammatory cytokines, leading to macrophage activation and the production of MMPs. Additionally, NF-κB regulates M1/M2 polarization of macrophages, and through granulocyte–macrophage colony-stimulating factor (GM-CSF), their differentiation into dendritic cells [100]. The increased activation of NF-κB leads to an excessive inflammatory response, the release of pro-inflammatory cytokines into the maternal circulation, and endothelial stress and contributes to the development of PE [100].

### 4.3. Angiogenesis and Imbalance between Angiogenic and Anti-Angiogenic Factors

The imbalance between angiogenic and anti-angiogenic factors is a crucial point in PE pathophysiology. Moreover, biomarkers of disturbed angiogenesis (mainly sFlt-1 and PlGF) are used in clinical practice for early PE screening, as well as for the prediction of PE complications in the mother and fetus. Chemerin involvement in the regulation of angiogenesis is quite well documented; however, this is not the case in PE. Chemerin stimulates angiogenesis via the CMLKR1 that is present on endothelial cells and dose-dependently induced MMP-2 and MMP-9 activity in these cells [26,101]. In a large family-based genetic epidemiological study, Bozaoglu et al. demonstrated that chemerin significantly mediated the formation of blood vessels to a similar extent to VEGF [29]. It is noteworthy that the chemerin-stimulating effect on angiogenesis was confirmed in both in vitro and in vivo models. Chemerin stimulated the differentiation of human umbilical vein endothelium cells (HUVECs) into capillary-like structures, functioning simultaneously as their chemoattractant via the MAPK and ERK1/2 signaling pathway. A similar effect was confirmed in a mouse corneal and rat aortic ring assays [101,102,103]. In contrast, Dhaou et al. demonstrated in mouse retinal hypoxia-driven neovascularization that chemerin may act as an anti-angiogenic factor [30]. The conflicting results of the studies may be associated with the used concentrations of bioactive chemerin that were higher than in the physiological condition, and the authors even highlight this problem as a limitation of their research [103]. 

The regulation of angiogenesis in PE has been extensively studied over recent years, leading to the identification of novel angiogenesis-related PE biomarkers, such as tissue inhibitor of metalloproteinase 3 (TIMP-3) [104]. As mentioned before, chemerin serum levels are elevated in pre-eclamptic patients. Basically, serum levels of anti-angiogenic biomarkers in PE, such as sFlt-1 or sEng, are also elevated [39,105,106]. Taking all these factors into consideration, we might suspect that chemerin in PE should have an anti-angiogenic rather than a pro-angiogenic effect; however, to date, there is no evidence for that; thus, this issue needs further investigation. Interestingly, in a recently published study, Ma et al. performed a bioinformatic analysis of angiogenesis-related genes and identified including 12 upregulated genes and 17 downregulated genes that are associated with PE; however, they were not related to chemerin and other adipokines, suggesting that the role of chemerin in PE development might be related to biological effects other than the regulation of angiogenesis [107].

### 4.4. Oxidative Stress and Endothelial Dysfunction

Endothelial dysfunction in PE is caused by multiple factors that are shed from the hypoxic placenta. Taking into consideration that both chemerin and its receptors are expressed in human placental tissue, in addition to the fact that there is evidence for enhanced chemerin secretion under hypoxia, we suggest that chemerin might be one of the molecules connecting the two stages of PE, leading in consequence to the onset of maternal symptoms, including hypertension, proteinuria, kidney and liver failure, and cerebrovascular events [7,108]. There are a number of mechanisms explaining the possible contribution of chemerin to endothelial dysfunction in PE. The first is that chemerin, by modulating glucose and lipid levels, affects lipid deposition in the endothelium. Both arteries and veins consist of three layers: endothelium, smooth muscle layer, and adventitia; however, perivascular adipose tissue (PVAT) was recently recognized as a tissue layer of its own with important functions in blood pressure regulation. CMLKR1 has been identified on the endothelial cells and vascular smooth muscle cells (VSMCc), as well as on vascular adipocytes [6,109,110]. Jia et al. demonstrated in an animal model that chemerin can increase lipid accumulation in atherosclerotic plaques and exacerbate plaque instability and also cause abnormal lipid accumulation in the livers and kidneys of model animals. These effects of chemerin are probably related to the p38/MAPK pathway [27]. In a recently published study, Tan et al. demonstrated in a mouse model that placental chemerin overexpression elevated maternal serum lipid level, leading to lipid accumulation in the placenta. Chemerin overexpression in this study was associated with increased levels of phospholipids, lysophospholipids, and cholesterol in both maternal blood and the placenta [111]. Lipid oxidation products (e.g., oxLDL) cause oxidative stress, endothelial dysfunction, and acute atherosclerosis [47,51]. Additionally, chemerin inhibits LDL uptake by decreasing LDLR and sortilin 1 (SORT1) in placental tissue. That leads to the enhanced release of lipids and lipid-related proteins (triglycerides, cholesterol, phospholipids, and chemerin) from the placenta into the maternal circulation. On the other hand, chemerin causes also a lower LDL uptake from the circulation to the placenta, and this contributes to dyslipidemia in the patient [111]. Gu et al. investigated the impact of chemerin overexpression on endothelial function, arterial stiffness, and early atherosclerosis in patients with hypertension and presented that a high chemerin serum level was an independent predictor of impaired endothelial function and increased arterial stiffness, even after adjustment for metabolic variables, inflammatory markers, and adipokines [112]. 

Multiple studies have demonstrated an association of chemerin with oxidative stress [113]. Turgut et al. presented that chemerin and free fatty acid serum levels were significantly higher in obese pre-eclamptic women in comparison to non-obese pre-eclamptic women and a normotensive control. Additionally, total antioxidant status was significantly lower, whereas total oxidative status was significantly higher in patients with PE compared to healthy controls [78]. Shen et al. observed that chemerin can induce autophagy in endothelial cells, which is associated with increased mitochondrial ROS generation and an activated 5’AMP-activated protein kinase α(p-AMPKα) signaling pathway. ROS are key signaling molecules that regulate angiogenesis and, thus, are essential for endothelial cell proliferation and migration. The authors suggested that chemerin induced ROS generation. In this study, the silencing of CMLKR1 significantly reduced ROS generation in human aorta endothelial cells and also inhibited the expression of beclin-1, a regulator of angiogenesis. Additionally, chemerin treatment resulted in an increase in AMPKα phosphorylation, negatively regulating the mammalian target of rapamycin (mTOR) signaling [114]. Yao et al. investigated the expression and function of chemerin and CMKLR1 system in the ovaries and granulosa cells of high-fat-diet-induced obese mice. They found that chemerin contributed to ROS accumulation and apoptosis through three signaling pathways: by activating NF-κB and AMPK signaling and inhibiting the AKT signaling pathway [115]. In another study, Bulut et al. revealed a significant positive correlation between plasma chemerin and Thioredoxin Reductase (TrxR) levels, as well as between salivary chemerin levels and sulfhydryl, indicating the association of chemerin with oxidative stress markers in patients with GDM [116]. Fulop et al. demonstrated that in the population of non-diabetic obese patients, oxLDL and hsCRP are the strongest predictors of chemerin serum level, and they concluded that chemerin is associated with chronic inflammation and oxidative stress in this group of patients, even in the absence of insulin resistance. Therefore, chemerin may be useful as a biomarker for increased cardiovascular risk and potentially one of the factors connecting PE with increased cardiovascular risk in later life [117]. 

Another important aspect is the impact of chemerin on NO signaling. Chemerin was found to reduce NO production, enhance NO breakdown, and also decrease NO-dependent cyclic guanosine monophosphate (cGMP) signaling, thereby reducing vascular relaxation [94]. That leads to an increase in ROS production in endothelial cells [6]. It was confirmed that in human aorta endothelial cells that were treated with chemerin, mitochondrial ROS production was increased, whereas the knockdown of CMLKR1 receptor caused a decrease in ROS production [114]. Chemerin stimulates Nox-derived ROS generation, leading to a pro-inflammatory response via MAPK activation in endothelial cells. Additionally, via redox-sensitive processes, chemerin regulates proliferation and apoptosis in VSMCs s [95]. Neves et al. demonstrated that the chemerin receptor blockade by CCX832 inhibits renal ROS production and protein oxidation. Potential mechanisms underlying this effect include the upregulation of Nox 4 (the most abundant Nox isoform in the kidney) and the downregulation of the nuclear erythroid-2-like factor (2Nrf2)-regulated anti-oxidant genes. These processes were observed in an animal model in the absence of renal structure injury or fibrosis, indicating that chemerin-induced renal dysfunction may occur early, preceding histological renal damage [118]. During PE, renal damage often occurs, and chemerin overexpression may thus contribute to renal impairment in pre-eclamptic patients and serve as a marker of early renal dysfunction, occurring before structural kidney damage. On the contrary, Wang et al. presented a study indicating that chemerin may play a protective role by regulating HUVEC-induced NO signaling in pre-eclampsia. They found that serum chemerin levels were increased in pre-eclampsia, while eNOS was decreased, and they negatively correlated with each other. Surprisingly, chemerin significantly and dose-dependently (in a range of 10–500 ng/mL) increased NO concentrations in HUVEC supernatants via the PI3K/Akt signaling pathway, suggesting that it has a protective effect on the endothelium [119]. It is noteworthy that the mean serum chemerin in severe PE patients in this study was 493.83 ± 105.23 ng/mL, so it was very close to the upper limit of the studied chemerin concentration influencing NO production in HUVEC [119]. What about higher levels of chemerin? It is widely known that chemerin may have quite opposite effects on different processes (pro- and anti-inflammatory, pro-angiogenic, and anti-angiogenic) depending on the context and disease, and it is possible that higher chemerin levels may inhibit NO production. In patients with PE in this study, the chemerin serum level was negatively correlated with eNOS level. We suggest that the effect of chemerin on endothelial cells in PE is enhanced by the overlapping of circulating chemerin (probably at least partially from placental origin) and local chemerin produced in PVAT. Other research has evidenced that the concentration of chemerin experienced by the vasculature is significantly higher than that which is circulating because of its production in PVAT, especially in patients with metabolic syndrome [109]. Metabolic syndrome is a cluster of cardiovascular disease risk factors, including obesity, atherogenic dyslipidemia, raised blood pressure, insulin resistance, and pro-inflammatory states, and women with metabolic syndrome are at an increased risk of both PE and GDM [120,121]. 

There is also evidence for a direct effect of chemerin on arterial contraction. A very interesting study that was performed by Watts et al. explained chemerin involvement in vasoconstriction in a rat model, indicating two different components in this process. First, chemerin directly causes contraction in the arteries without PVAT, probably due to the elevation of intracellular calcium. Second, in arteries containing PVAT, chemerin may participate in arterial contraction initiated by other vasoconstrictors (prostaglandin F2alpha, phenylephrine, and KCl in this study) [108]. In other studies, chemerin potentiated also the pro-contractile effect of endothelin-1 [122,123]. Additionally, it was demonstrated that different isoforms of chemerin elicit different second messenger signaling through the same receptor, e.g., chemerin 9 but not recombinant full-length chemerin elevates intracellular calcium in a manner that is antagonized by CCX832 [109]. It has been confirmed that PVAT is present in uterine arteries and plays an important role in the uterine blood flow regulation, especially in pregnancy. Interestingly, in vivo uterine PVAT potentiates uterine artery blood flow in pregnant rats, whereas in isolated preparations, uterine PVAT has pro-contractile and anti-dilatory effects on uterine arteries [124]. Another mechanism contributing to blood pressure control is that chemerin also regulates the proliferation and migration of VSMCs, as it is responsible not only for vascular dysfunction, but also abnormal vascular structure. Studies have shown that the short-term in vitro treatment of VSMCs (20 min) increased the proliferation and migration capacity of VSMCs via MAPK and Akt/ERK signaling, whereas prolonged incubation with chemerin (6 h) led to VSMCs apoptosis [95,125,126,127]. Other research indicates that chemerin can also induce VSMCs dysfunction by augmenting oxidative stress and promoting inflammation, because increased ROS accumulation and elevated expression of IL-1β, IL-6, and monocyte chemoattractant protein-1 (MCP-1) were detected in chemerin-treated VSMCs [6,95,128]. Finally, PVAT consists not only of adipocytes, but also other cells, e.g., sympathetic nerves can also be found in this layer. An experimental rat model revealed that chemerin may enhance sympathetic nerve function, because it potentiates electrical-field-stimulation-induced arterial contraction [110,129]. 

## 5. Important Implications of Chemerin Involvement in PE Pathophysiology: Early vs. Late-Onset PE and Future Cardiovascular Risk

The evidence presented in this review indicates that chemerin plays an important role in the pathophysiology of PE. There still remains a question about the differences in chemerin involvement between early- and late-onset PE. It is known that there are essential distinctions between these subtypes of PE. Early-onset disease is associated mainly with abnormal uterine artery remodeling, leading to placental malperfusion, and is accompanied very often by FGR. Late-onset PE is related to the STB stress caused by mismatch between normal maternal perfusion and the metabolic demands of the placenta and fetus, accompanied by cellular senescence of the ageing placenta [33,34,38]. The vast majority of studies concerning chemerin expression in PE do not separate early-onset and late-onset groups. Bartho et al. in a recently published study presented that chemerin serum levels, as well as its placental expression, were increased in women with early-onset disease. Additionally, in vitro gene studies showed that under hypoxia, RARRES2 gene expression was increased in STB [130]. These results reinforce the hypothesis that chemerin production in placental tissue is enhanced in hypoxic conditions and contributes to PE development. Interestingly, in the same study, the authors demonstrated increased circulating chemerin levels at 36 weeks of gestation in normotensive women who later developed PE (late-onset disease). Thus, they concluded that in both PE subtypes, the main chemerin source is placenta [130]. This study demonstrates that chemerin is involved in the development of both PE subtypes, probably because it is closely related to STB stress. Moreover, taking it together with the results of another study demonstrating elevated first-trimester maternal serum chemerin levels in women who developed PE compared with those without PE and also in patients who developed severe PE compared with mild PE, we suggest that chemerin overexpression in pregnancy is the cause rather than the consequence of PE because elevated chemerin blood concentrations are present long before the onset of clinical symptoms of the disease [75]. This phenomenon was also confirmed in animal models, in which chemerin placental overexpression caused by gene manipulations led to the development of PE-like symptoms [7].

Another important aspect of PE pathophysiology is the elevated risk of cardiovascular disease in future life. Multiple studies have demonstrated that PE is associated with significantly increased risk of heart failure, coronary artery disease, stroke, and cardiovascular death later in life [131,132,133]. It is unclear if PE is an independent risk factor for cardiovascular incidents in the future or if pregnancy is only a trigger of cardiovascular alterations that were present earlier but did not manifest with clinical symptoms. Chen et al. investigated the association between third-trimester maternal chemerin serum levels in PE and blood pressure levels after delivery (in a mean follow-up time of 2.8 years). They found that serum chemerin levels were significantly elevated in pre-eclamptic women in comparison to healthy controls. The incidence of postpartum hypertension in patients with PE was 45%, whereas in the control group, it was 17.4%. Maternal third-trimester chemerin serum levels positively correlated with postpartum blood pressure, and the authors concluded that chemerin may serve as a marker for the early prediction of postpartum hypertension [134]. Together with the results of another study confirming elevated chemerin serum levels 6 months after delivery in former PE patients in comparison to healthy controls [73], it is possible that chemerin might be one of the factors linking PE and future cardiovascular risk, but it needs further research.

## 6. Summary

**Figure 1:**
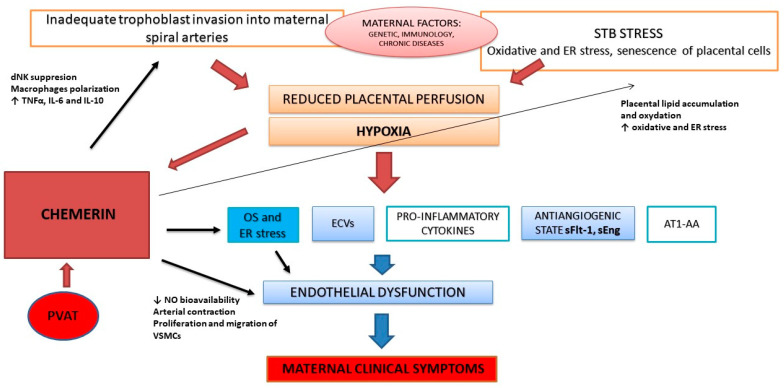
The potential role of chemerin in the pathophysiology of PE.

In this review, we summarize the current state of knowledge about the potential role of chemerin in PE pathophysiology. The available research demonstrates that chemerin is involved in different biological processes that are important during PE development, including oxidative stress, inflammation, angiogenesis, and endothelial dysfunction. Multiple studies have shown chemerin overexpression in placental tissue, as well as elevated serum chemerin levels in women with PE. We suggest that chemerin is one of the factors that are shed from the hypoxic placenta and connect two disease stages, causing maternal endothelial dysfunction in two different ways: 1. indirectly, by enhancing oxidative stress and reducing NO availability, and 2. directly, by acting as a vasoconstrictor. Chemerin may also significantly affect the first stage of PE development by enhancing oxidative and ER stress in trophoblasts and influencing the decidual NK cells that are responsible for uterine artery remodeling. The potential role of chemerin in PE pathophysiology is summarized in Figure 1. Further research is needed to confirm the exact mechanisms of the action of chemerin in PE and enable the elaboration of new strategies for prevention and therapy dedicated to women with a high risk of PE.

PVAT—perivascular adipose tissue; STB—syncytiotrophoblast; OS—oxidative stress; ER—endoplasmic reticulum; ECVs—extracellular vesicles; sFlt-1—soluble fms-like tyrosine kinase 1; sEng—soluble endoglin; AT1-AA angiotensin II 1 receptor autoantibodies; TNFα—tumor necrosis factor α; VSMCs—vascular smooth muscle cells.
